# Shanz-assisted Closed Reduction (SACR): A Novel Reduction Technique for Elastic Stable Intramedullary Nailing of Pediatric Forearm Fractures

**DOI:** 10.1055/s-0044-1787548

**Published:** 2024-07-18

**Authors:** Julio Javier Masquijo

**Affiliations:** 1 Departamento de Ortopedia Infantil, Sanatorio Allende, Córdoba, Argentina

**Keywords:** child, forearm injuries, fracture fixation, intramedullary, radius fractures

## Abstract

Forearm fractures involving both bones are prevalent among pediatric patients. Elastic stable intramedullary nailing (ESIN) is an excellent option for forearm fractures that require surgical stabilization in children and adolescents. Proximal third fractures can be particularly challenging to reduce using closed techniques, and multiple unsuccessful attempts at nail insertion can increase the risk of compartment syndrome. In this article, we present a novel technique called Shanz-assisted closed reduction (SACR), which involves using a 4-mm Shanz to facilitate alignment and nail insertion while avoiding complications associated with longer operative times and open reduction.

## Introduction

Both-bone forearm fractures are among the most common pediatric fractures, accounting for approximately 40% of all cases. 1 Treatment typically involves closed reduction and casting, but surgical intervention may be necessary for significantly displaced, highly comminuted, or segmental fractures. Other surgical indications include open fractures, associated neurovascular injury, fractures refractory to closed reduction and casting, and concomitant ipsilateral humerus fractures (also known as floating elbow). 2 Elastic stable intramedullary nailing (ESIN) is an excellent option for forearm fractures in children and adolescents due to its minimally invasive approach. However, proximal third fractures can be particularly challenging to reduce using closed techniques, and multiple unsuccessful attempts at nail insertion can increase the risk of compartment syndrome. 3 If closed reduction is unsuccessful, open reduction with internal fixation (ORIF) may be necessary.

This article describes a novel technique using a 4-mm Shanz to facilitate nail insertion and avoid complications associated with longer operative times and open reduction (OR).

## Technical Description

**Fig. 1:**
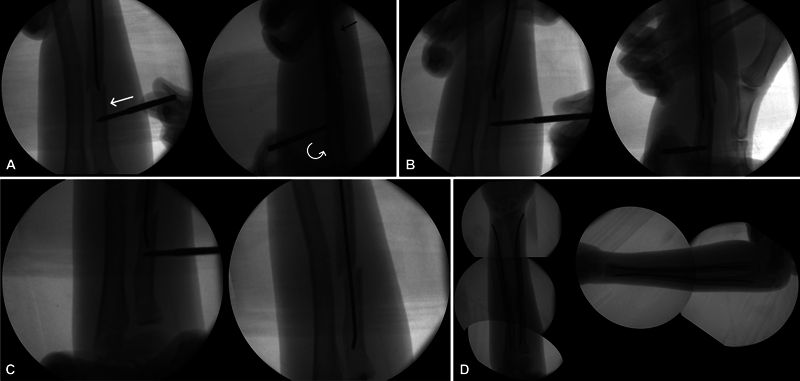
A) The flexible nail is inserted under fluoroscopic guidance up to the fracture site. A monocortical 4-mm Schanz pin is placed three centimeters proximal to the fracture site. A drill sleeve is used to protect soft tissues. The reduction maneuver involves translation of the fragment in the coronal plane, pronation of the proximal fragment, and supination of the distal segment. B) The elastic nail is advanced to reach the proximal fragment. C) The Schanz pin is removed. D) Final construct.

The patient is positioned in the supine position and given general anesthesia. The starting point for the radial nail is marked between the first and second dorsal compartments, 1 cm proximal to the physis. The ulnar starting point is marked on the posterior border of the ulna, distal to the physis. The radial bone is typically addressed first as reduction may be difficult after fixation of the ulna. Dissection is performed between the first and second dorsal extensor compartments with care taken to protect the superficial radial nerve. An opening is made in the distal radius cortex proximal to the physis, and a flexible nail is inserted under fluoroscopic guidance. The arm is manipulated to reduce the fracture. If unsuccessful after three attempts, the Shanz-assisted closed reduction (SACR) technique is utilized ( Fig. 1 ).

**Fig. 2:**
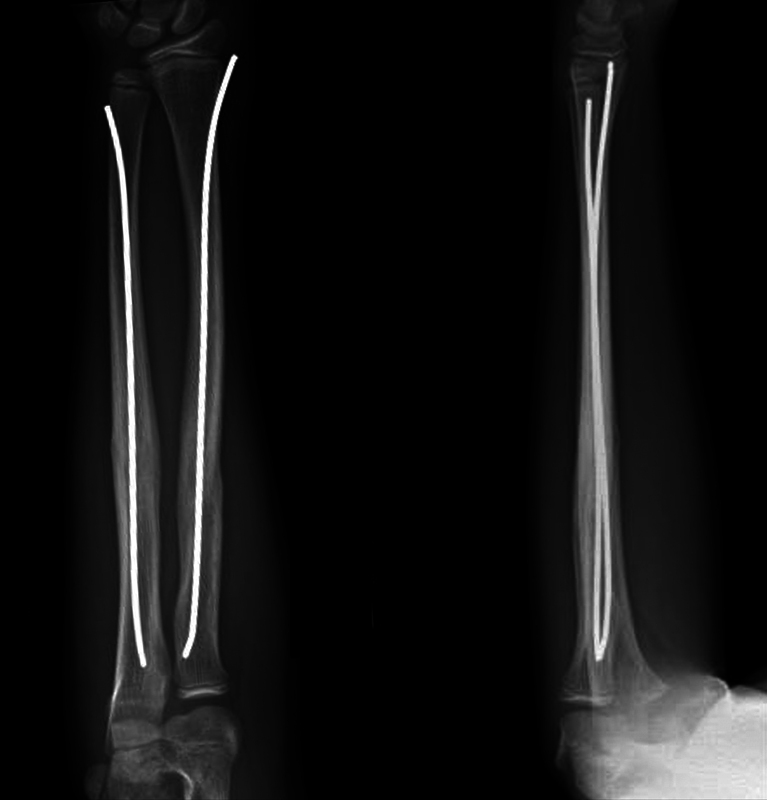
Anteroposterior and lateral radiograph at 6 months follow-up.

For the SACR technique, a 1- to 1.5 cm skin incision is made and gradually deepened using forceps and retractors until the bone is reached. A 4-mm Schanz pin is placed 3 cm proximal to the fracture site through a drill sleeve to protect soft tissues. A monocortical pin is used to manipulate the proximal fragments, allowing the surgeon to correct rotation, angulation, and translation until the elastic nail can be inserted. In proximal third forearm fractures, the supinator and biceps will tend to supinate the proximal segment, so the reduction maneuver involves pronation of the proximal fragment and supination of the distal segment. Once the elastic nail is inside the proximal fragment, the Schanz pin is removed. After addressing the radial bone, attention is turned to fixation of the ulna. Although the technique can also be used in the ulna, it is rarely required. An opening is made in the proximal ulna, and a flexible nail is inserted. Both nails (radius and ulna) are impacted to 1 cm short of the expected ending point, then cut flush to the skin and impacted an additional 1 cm below the skin. Postoperatively, the patient is immobilized in a long arm splint for 2 weeks, followed by full arm motion. Radiographs are taken at 6-week intervals to assess union, and the nails are typically removed at 9 to 12 months ( Fig. 2 ).

## Discussion

Elastic intramedullary nailing of pediatric forearm fractures represents a popular technique to treat unstable fractures that fail closed reduction and casting in children. Surgical stabilization with ESIN fixation is minimally invasive, provides excellent alignment, facilitates postoperative care, and eliminates concerns about acceptable alignment. 4

However, certain fracture patterns, such as those in the proximal third of the radius and ulna or comminuted midshaft, may require OR to facilitate nail insertion. Multiple attempts at closed reduction may increase the risk of compartment syndrome, which has been associated with longer operative times and increased intraoperative fluoroscopy burden. 2 5 For this reason, most pediatric orthopedic surgeons suggest performing OR after three unsuccessful attempts at closed reduction. 6 Other authors have proposed a Kapandji-like technique to avoid OR. 7 Using fluoroscopy, a 2.0-mm Kirschner wire can be inserted at the fracture site to elevate and reduce the fragments. Disadvantages of this technique are the risk of neurovascular injury and infection associated with multiple attempts.

## Final Considerations

We describe a simple and reproducible surgical tip to ease nail insertion and avoid complications related with longer operative times and OR. While forearm proximal pin placement is generally associated with low morbidity, it is essential to consider the potential risks to the radial nerve, particularly the posterior interosseous nerve (PIN). The PIN is a branch of the radial nerve that originates from the radial nerve at the radiohumeral joint line. It then travels along the posterior aspect of the forearm, passing through the supinator muscle at the arcade of Frohse. Continuing its course, the nerve winds around the radial neck within the substance of the muscle, eventually reaching the posterior compartment of the forearm and the interosseous membrane of the forearm. It finally terminates as a source of sensation to the dorsal wrist capsule. Given the proximity of the PIN to the surgical site during forearm proximal pin placement, a thorough understanding of the forearm's anatomy is crucial to ensure safe and precise pin insertion. To minimize the risk of PIN injury during the procedure, surgeons utilize careful dissection techniques and employ a drill sleeve to safeguard surrounding soft tissues. The same principle can be applied to other long bones, like the femur, tibia, and humerus, that require ESIN. We have also used 4-mm Shanz to assist reduction in supracondylar fractures of the humerus to enable closed reduction and percutaneous pinning. In the forearm, the SACR technique allows a more effective manipulation of the proximal fragment to restore alignment and facilitate successful nailing in pediatric patients.
