# Supplementary material for: Shanz-assisted Closed Reduction (SACR): A Novel Reduction Technique for Elastic Stable Intramedullary Nailing of Pediatric Forearm Fractures
Source: Rev Bras Ortop (Sao Paulo). 2024 Jul 18;59(5):e786–8. doi: 10.1055/s-0044-1787548 (PMC11624929; doi:10.1055/s-0044-1787548)
Supplement: Related texts

# Research article: Redução fechada assistida por Schanz (SACR): Uma nova técnica de redução para fixação intramedular elástica estável de fraturas pediátricas do antebraço

**Keywords:** criança, fixação intramedular de fraturas, fraturas do rádio, lesões do antebraço

## Abstract

Fraturas dos dois ossos do antebraço são prevalentes entre pacientes pediátricos. A haste intramedular elástica estável (ESIN, na sigla em inglês) é uma excelente opção para fraturas do antebraço que requerem estabilização cirúrgica em crianças e adolescentes. A redução fechada de fraturas do terço proximal pode ser particularmente difícil, e as múltiplas tentativas malsucedidas de inserção da haste podem aumentar o risco de síndrome compartimental. Este artigo apresenta uma nova técnica chamada redução fechada assistida por Schanz (SACR, na sigla em inglês) com uso de um Shanz de 4 mm para facilitar o alinhamento e a inserção da haste e evitar as complicações associadas a tempos operatórios mais longos e à redução aberta.

## Introdução

As fraturas dos dois ossos do antebraço estão entre as fraturas pediátricas mais comuns, representando aproximadamente 40% de todos os casos. 1 O tratamento normalmente requer redução fechada e moldagem, mas a intervenção cirúrgica pode ser necessária em fraturas com luxações significativas, altamente cominutivas ou segmentares. Outras indicações cirúrgicas incluem fraturas expostas, lesão neurovascular associada, fraturas refratárias à redução fechada e gesso e fraturas concomitantes do úmero ipsilateral (também conhecidas como cotovelo flutuante). 2 A haste intramedular elástica estável (ESIN, na sigla em inglês) é uma excelente opção para fraturas do antebraço em crianças e adolescentes devido à sua abordagem minimamente invasiva. No entanto, a redução fechada de fraturas do terço proximal pode ser particularmente difícil, e as múltiplas tentativas malsucedidas de inserção da haste podem aumentar o risco de síndrome compartimental. 3 Em caso de insucesso da redução fechada, a redução aberta com fixação interna (RAFI) pode ser necessária.

Este artigo descreve uma nova técnica utilizando uma haste de 4 mm para facilitar a inserção da haste e evitar as complicações associadas a tempos operatórios mais longos e à redução aberta (RA).

## Descrição da técnica

O paciente é colocado em decúbito dorsal e recebe anestesia geral. O ponto inicial da haste radial é marcado entre o primeiro e o segundo compartimentos dorsais, 1 cm proximal à fise. O ponto inicial ulnar é marcado na borda posterior da ulna, distal à fise. De modo geral, o osso radial é abordado primeiro, pois sua redução pode ser difícil após a fixação da ulna. A dissecção é realizada entre o primeiro e o segundo compartimentos extensores dorsais com cuidado para proteger o nervo radial superficial. Uma abertura é feita no córtex distal do rádio, proximal à fise, e uma haste flexível é inserida sob orientação fluoroscópica. O braço é manipulado para reduzir a fratura. Na ausência de sucesso após três tentativas, a técnica de redução fechada assistida por Schanz (SACR, na sigla em inglês) é utilizada ( Fig. 1 ).

Para a técnica SACR, faz-se uma incisão na pele de 1 a 1,5 cm que é aprofundada gradativamente com pinças e afastadores até atingir o osso. Um pino de Schanz de 4 mm é colocado 3 cm proximal ao sítio da fratura com uma bucha de perfuração para proteger os tecidos moles. Um pino monocortical é utilizado para manipular os fragmentos proximais, permitindo que o cirurgião corrija a rotação, angulação e translação até que a haste elástica possa ser inserida. Nas fraturas do terço proximal do antebraço, o supinador e o bíceps tenderão a supinar o segmento proximal; logo, a manobra de redução requer pronação do fragmento proximal e supinação do segmento distal. Com a haste elástica no interior do fragmento proximal, o pino de Schanz é removido. Após a abordagem ao osso radial, a atenção se volta para a fixação da ulna. Embora a técnica também possa ser usada na ulna, raramente é necessária. É feita uma abertura na ulna proximal e uma haste flexível é inserida. Os dois pinos (no rádio e na ulna) são impactados a 1 cm do ponto final esperado, depois cortados rente à pele e impactados mais 1 cm abaixo da pele. No período pós-operatório, o paciente é imobilizado com tala longa no braço por 2 semanas e, a seguir, a movimentação do braço pode ser completa. As radiografias são obtidas em intervalos de 6 semanas para avaliação da consolidação e os pinos são normalmente removidos entre 9 e 12 meses ( Fig. 2 ).

## Discussão

A haste intramedular elástica em fraturas pediátricas do antebraço é uma técnica popular para tratamento de fraturas instáveis com insucesso à redução fechada e ao gesso em crianças. A estabilização cirúrgica por fixação com ESIN é minimamente invasiva, proporciona excelente alinhamento, facilita o cuidado pós-operatório e elimina preocupações acerca do alinhamento aceitável. 4

No entanto, certos padrões de fratura, como aqueles no terço proximal do rádio e ulna ou diáfise cominutiva, podem exigir RA para facilitar a inserção da haste. Múltiplas tentativas de redução fechada podem aumentar o risco de síndrome compartimental, que tem sido associada a tempos operatórios mais longos e aumento da carga de fluoroscopia intraoperatória. 2 5 Por esse motivo, a maioria dos cirurgiões ortopédicos pediátricos sugere a realização da cirurgia após três tentativas malsucedidas de redução fechada. 6 Outros autores propuseram uma técnica semelhante à de Kapandji para evitar a RA. 7 Usando fluoroscopia, um fio de Kirschner de 2,0 mm pode ser inserido no sítio de fratura para elevar e reduzir os fragmentos. As desvantagens dessa técnica são o risco de lesão neurovascular e infecção associada às múltiplas tentativas.

## Considerações finais

Este artigo descreve um procedimento cirúrgico simples e reprodutível para facilitar a inserção da haste e evitar complicações relacionadas a tempos operatórios mais longos e à RA. Embora a colocação do pino proximal no antebraço esteja geralmente associada à baixa morbidade, é essencial considerar os possíveis riscos para o nervo radial, particularmente o nervo interósseo posterior (PIN). O PIN é um ramo do nervo radial que se origina na linha articular radioumeral. Em seguida, percorre a face posterior do antebraço, passando pelo músculo supinador na arcada de Frohse. Continuando seu curso, o nervo serpenteia ao redor do colo radial no interior do músculo, chegando ao compartimento posterior do antebraço e à membrana interóssea do antebraço. Por fim, termina como uma fonte de sensibilidade para a cápsula dorsal do punho. Dada a proximidade entre o PIN e o sítio cirúrgico durante a colocação do pino proximal no antebraço, uma compreensão completa da anatomia do antebraço é crucial para garantir a inserção segura e precisa do pino. Para minimizar o risco de lesão do PIN durante o procedimento, os cirurgiões utilizam técnicas cuidadosas de dissecção e uma manga de perfuração para proteger os tecidos moles circundantes. O mesmo princípio pode ser aplicado a outros ossos longos, como fêmur, tíbia e úmero, que requerem ESIN. Também utilizamos Shanz de 4 mm para auxiliar a redução de fraturas supracondilares do úmero para permitir a redução fechada e fixação percutânea. No antebraço, a técnica SACR permite uma manipulação mais eficaz do fragmento proximal para restaurar o alinhamento e facilitar a fixação bem-sucedida em pacientes pediátricos.

**Fig. 1:** A haste flexível é inserida sob orientação fluoroscópica até o local da fratura. Um pino de Schanz monocortical de 4 mm é colocado três centímetros proximal ao sítio da fratura. Uma manga de perfuração é usada para proteger os tecidos moles. A manobra de redução envolve a translação do fragmento no plano coronal, a pronação do fragmento proximal e a supinação do segmento distal. B) A haste elástica é avançada até atingir o fragmento proximal. C) O pino de Schanz é removido. D) Construto final.

**Fig. 2:** Radiografia anteroposterior e em perfil aos 6 meses acompanhamento.
